# Supplementary material for: Dual-Action Gemcitabine Delivery: Chitosan–Magnetite–Zeolite Capsules for Targeted Cancer Therapy and Antibacterial Defense
Source: Gels. 2024 Oct 21;10(10):672. doi: 10.3390/gels10100672 (PMC11507657; doi:10.3390/gels10100672)
Supplement: Supplementary file 1 [file gels-10-00672-s001.zip › gels-3226074-supplementary.pdf]

Supplementary Material

Gels

Dual-Action Gemcitabine Delivery: Chitosan–Magnetite–Zeolite Capsules for Targeted Cancer Therapy and Antibacterial Defense

Yuly Andrea Guarín-González [1,*], Gerardo Cabello-Guzmán [2], José Reyes-Gasga [3], Yanko Moreno-Navarro [4], Luis Vergara-González [5], Antonia Martin-Martín [6], Rodrigo López-Muñoz [6], Galo Cárdenas-Triviño [1,†] and Luis F. Barraza [7,*]

* Corresponding authors.
E-mail address: yguarin@ubiobio.cl (Y.A.G.-G.); luis.barraza@uss.cl (L.F.B.)
† Deceased author

Table. S1 Phases quantification of zeolite and magnetite

**Zeolite**			
**Phase**	**Mineral Phase**	**Formula**	**Quantification**
**1**	Clinoptilolite – Ca	$KNa_2Ca_2(Si_{29}A_{17})O_{72} \cdot 24H_2O$	45.08 %
**2**	Clinoptilolite – Na	$(Na,K,Ca)_5Al_6Si_{30}O_{72} \cdot 18H_2O$	46.62 %
**3**	Barrerite	$(Na,K,Ca)_2(Si,A)_9O_{18} \cdot 7H_2O$	2.94 %
**4**	Graphite	C	5.35 %
**Magnetite**			
**Phase**	**Mineral Phase**	**Formula**	**Quantification**
**1**	Magnetite	$Fe + 2Fe_2 + 3O_4$	70.04 %
**2**	Maghemite	Fe_2O_3	29.80 %
**3**	Iron oxide and titanium hydride	Eta- $Ti_2FeO_{0.2}H_{2.8}$	0.16 %

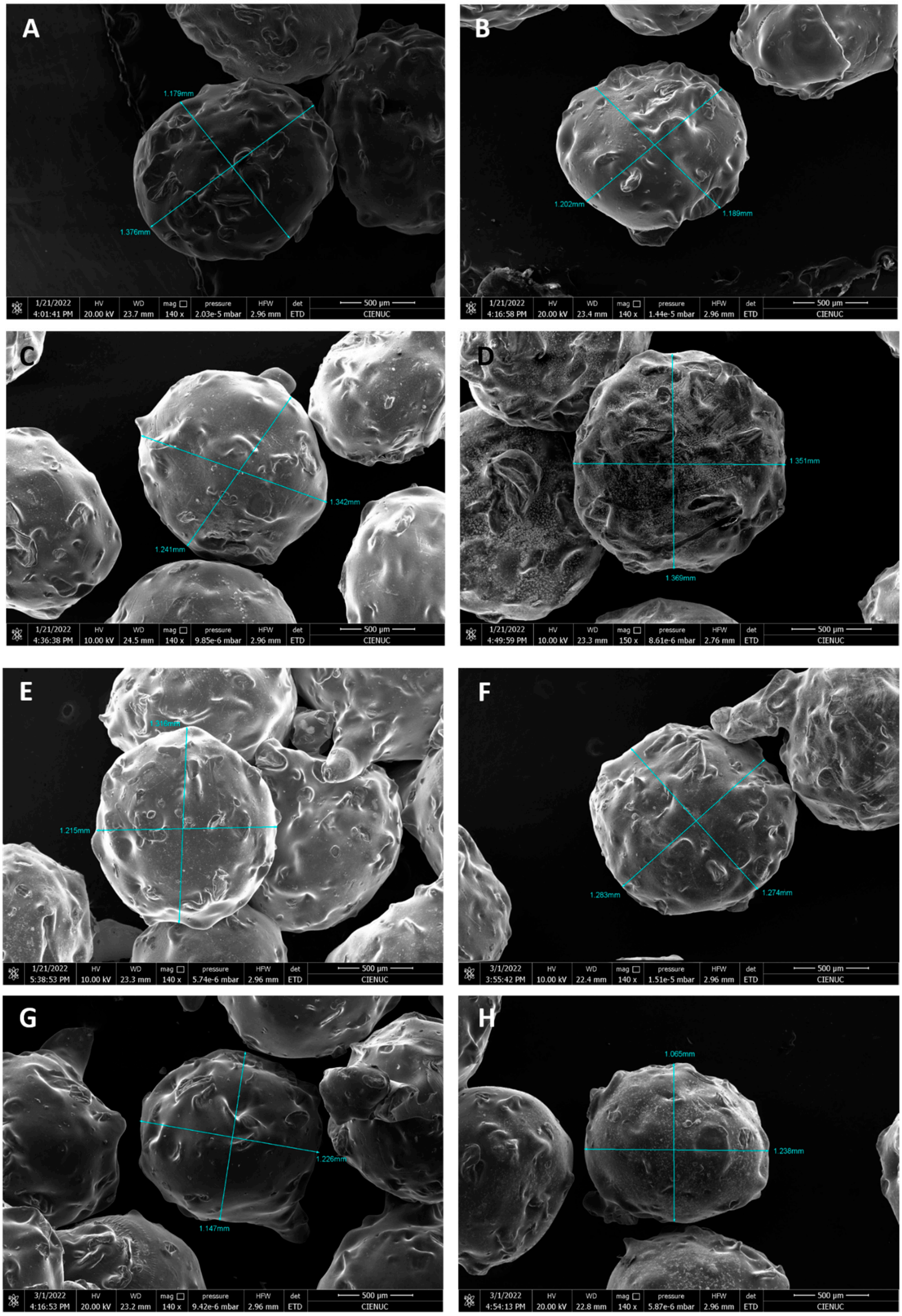
A
1.179mm
1.376mm
B
1.202mm
1.189mm
C
1.342mm
1.241mm
D
1.351mm
1.369mm
E
1.215mm
F
1.283mm
1.274mm
G
1.226mm
1.147mm
H
1.065mm
1.238mm
500 µm
CIENUC

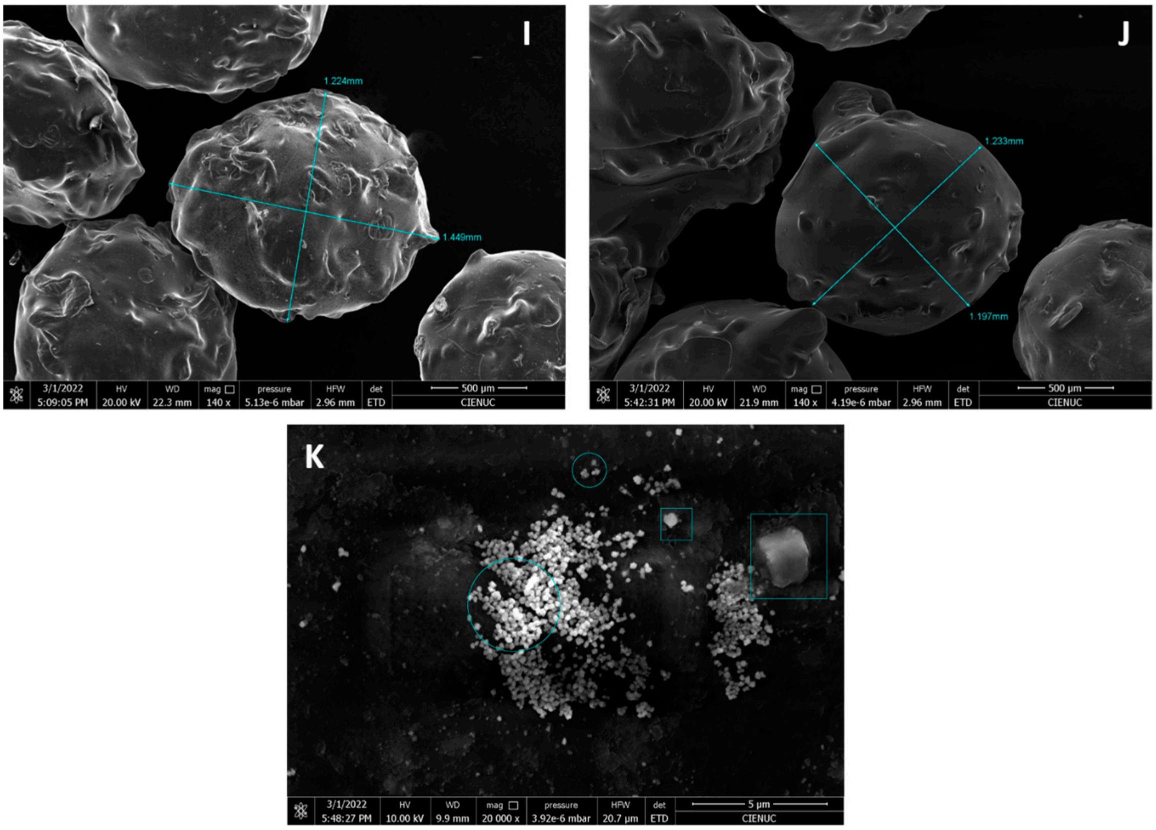

Figure S1. FESEM micrographs of macrocapsules; (A) group 3, (B) group 4, (C) group 5, (D) group 6, (E) group 8, (F) group 9, (G) group 10, (H) group 11, (I) group 12, (J) group 14 and (K) minerals in the surface focus of group 14.

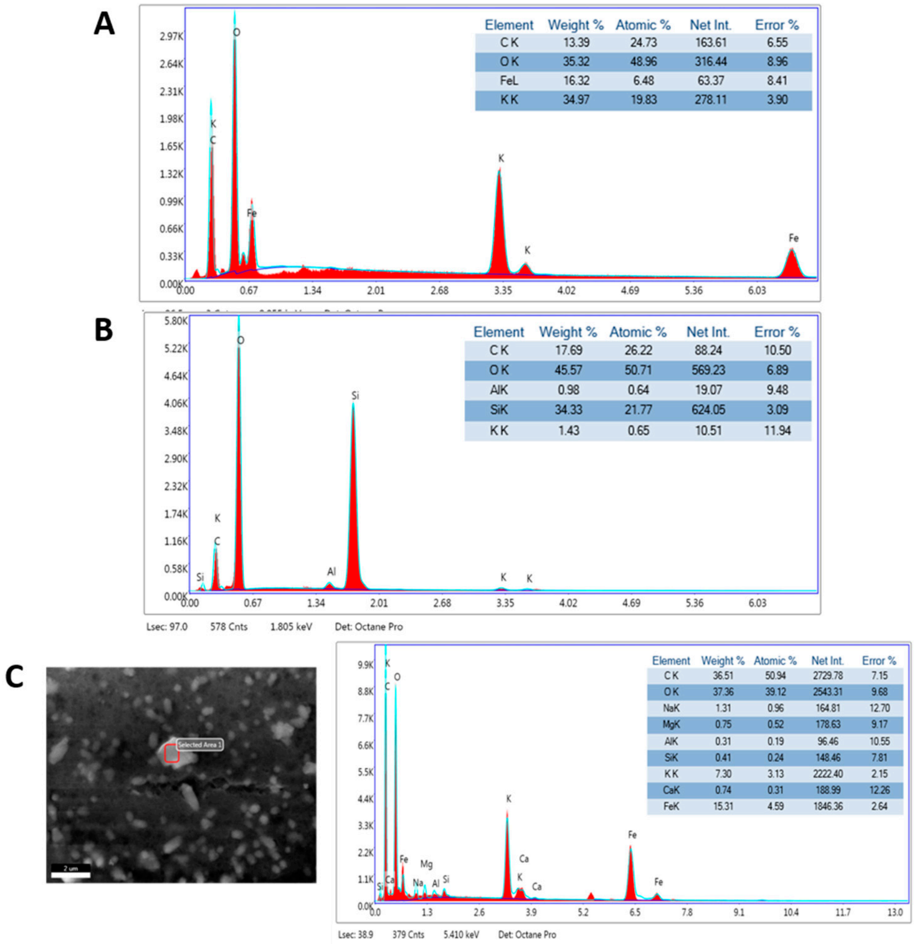

Figure S2: EDS analysis of the group 5 (A), group 7 (B) and group 14 (C)

Table S2: Characteristic bands of macrocapsule groups 3, 5 and 7, for identification of components interaction.

Group	Sample	IR- absorption band (cm^{-1})	Movement description	Bond
3	Chitosan 4% + **Gemcitabine 0.0025%**	3294	Stretch vibration	-OH
		1669	Amide I Axial deformation	-C=O
		1428	Deformation	CH_3
		1157	Antisymmetrical stretch vibration	C-O-H
		1091	Symmetrical vibration	C-O-C
		**3541**	**Primary amine stretching**	**NH_2**
		**1386**	**Stretch vibration**	**C-F**
5	Chitosan 4% + **Magnetite 0.1%**	3152	Stretch vibration	-OH
		1675	Amide I Axial deformation	-C=O
		1584	Torsion	NH_2
		1426	Deformation	CH_3
		1158	Antisymmetrical stretch vibration	C-O-H
		**561**	**Stretch vibration**	**Fe-O**
7		1665	Amide I Axial deformation	-C=O

	1596	Torsion	NH_2
	1425	Deformation	CH_3
	1259	Symmetrical stretch vibration	C-O-H
	1157	Antisymmetrical stretch vibration	C-O-H
	1077	Symmetrical vibration	C-O-C
Chitosan 4% + **Zeolite 0.1%**	**3511**	**Stretching vibration acid hydroxyls**	**Si-O(H)-Al**
	**3291**	**Tensile vibration**	**-OH (zeolite) and –NH_2 (chitosan)**
	**1035**	**Asymmetric valence of the tetrahedron**	**SiO_4**
	**487**	**Bending vibration**	**Si-O-Si**

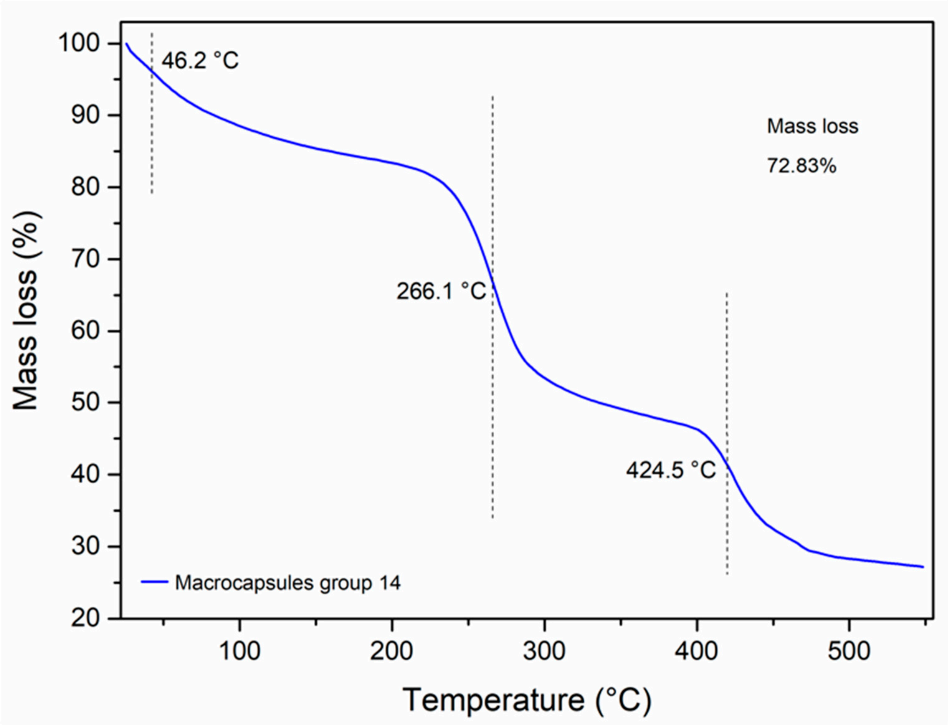

FigureS3: Thermogram, macrocapsules group 14

Table S3: Estimated GEM concentrations (mg/mL) from extracts.

Group	Extract (%)	[GEM mg/mL]
	100	0.0225
	80	0.0180
G3	60	0.0135

	40	$9.00x10^{-3}$
	20	$4.50x10^{-3}$
G10	100	0.0202
	80	0.0161
	60	0.0121
	40	$8.06x10^{-3}$
	20	$4.03x10^{-3}$
G11	100	0.0211
	80	0.0168
	60	0.0126
	40	$8.42x10^{-3}$
	20	$4.21x10^{-3}$
G14	100	0.0208
	80	0.0166
	60	0.0125
	40	$8.00x10^{-3}$
	20	$4.00 x10^{-3}$
